# Measurement of intrinsic optical backscattering characteristics of cells using fiber-guided near infrared light

**DOI:** 10.1186/1475-925X-9-12

**Published:** 2010-02-25

**Authors:** Ching-Huang Hsu, Gwo-Ching Chang, En-Ting Li, Yu-Jing Lin, Jia-Jin Jason Chen

**Affiliations:** 1 Institute of Biomedical Engineering, National Cheng Kung University, Tainan, 701 Taiwan, ROC; 2 Department of Information Engineering, I-Shou University, Kaohsiung, 840 Taiwan, ROC

## Abstract

**Background:**

Intrinsic optical signals (IOS), which reflect changes in transmittance and scattering light, have been applied to characterize the physiological conditions of target biological tissues. Backscattering approaches allow mounting of the source and detector on the same side of a sample which creates a more compact physical layout of device. This study presents a compact backscattering design using fiber-optic guided near-infrared (NIR) light to measure the amplitude and phase changes of IOS under different osmotic challenges.

**Methods:**

High-frequency intensity-modulated light was guided via optic fiber, which was controlled by micromanipulator to closely aim at a minimum cluster of cortical neurons. Several factors including the probe design, wavelength selection, optimal measuring distance between the fiber-optical probe and cells were considered. Our experimental setup was tested in cultured cells to observe the relationship between the changes in backscattered NIR light and cellular IOS, which is believed mainly caused by cell volume changes in hypo/hyperosmotic solutions (± 20, ± 40 and ± 60 mOsm).

**Results:**

The critical parameters of the current setup including the optimal measuring distance from fiber-optical probe to target tissue and the linear relationship between backscattering intensity and cell volume were determined. The backscattering intensity was found to be inversely proportional to osmotic changes. However, the phase shift exhibited a nonlinear feature and reached a plateau at hyperosmotic solution.

**Conclusions:**

Our study indicated that the backscattering NIR light guided by fiber-optical probe makes it a potential alternative for continuous observation of intrinsic optical properties of cell culture under varied physical or chemical challenges.

## Background

Optoelectronic technologies have been applied in medical diagnosis and clinical monitoring to reveal various tissue characteristics by comparing the light emitted by a source with the light after it has interacted with a target [1-4]. Photons traveling in biological tissue undergo two main effects, scattering and absorption, which are known to depend on the properties and physiological conditions of biological tissues. Tissue absorption can be characterized by the intensity of incident photons diminishing along the observation path as photons collide with strong absorbers. Tissue scattering properties result mainly from a series of optical reflections and refractions of light after interaction with biological particles, droplets and fluid density fluctuations [2,4,5]. Based on these two optical properties, various noninvasive optical measurements for biostatic detection have been developed [3,6-9]. However, light absorption techniques rely mainly on strong absorbers or contrast agents which may not always exist in cellular tissues. In contrast to absorption methods, scattering behavior dominates the light propagation in cellular tissues.

The changes in light transmittance and scattering, commonly called intrinsic optical signal (IOS), have been applied to revealing various molecular, biochemical and morphological characteristics of cellular tissue [8-10]. At the cellular level, it is believed that IOS originates from changes in the refractive indexes of cytoplasm and extracellular matrix, which have been correlated with cell membrane potential, cell swelling and other cellular behavior under various physiological conditions [11]. Based on the onset of optical response, the fast scattering type, i.e. response in the millisecond range, was presumed to be caused by ionic flow across ion channels causing change in the refractive index of the cell membrane [9,12-14]. An almost synchronized response pattern between optical and electrical recordings was observed, indicating that optical methods could be used as an alternative approach for sensing cell membrane potentials [8,9,15,16]. The slow type of optical response occurred over the range of seconds and correlated with physical changes in the shape of a cell such as slow swelling or shrinking. Changes in cell volume or opacity were found to alter the intensities of transmitted and reflected light such that optical methods could be adopted for continuous monitoring of cell states [15,16] including the swelling of neuronal soma, astrocytes of rat brain slice under oxygen-glucose-deprived condition [10] and volume change under potassium treatment or electrical stimulation [17].

Two general detection schemes have been developed for light scattering, i.e. the forward and backward light scattering techniques. In biologic media, scattering is typically highly forward-directed (anisotropic). To achieve higher probability of collecting scattered photons, the most common biological light scattering studies employ designs which locate the detector on the far side of the target. However, forward scattering approaches require a complicated lens or alignment system such as a scanning microscope for precise alignment between the focused light beam and the target tissue at a specific angle in order to restrict receiving only the forward-scattered light of interest [8,9,18-20]. On the other hand, the backscattering approach requires an ultra-sensitive detection system to pick up the tiny amounts of backscattering photons. Nevertheless, the same-side arrangement of light source and optical detector in the backscattering approach can minimize the size of the sensing apparatus, which is more feasible for the design of advanced *in vivo *measuring systems. Several backscattering approaches with different probe designs such as optical fiber, gradient-index lens and nano-probe have been applied to measure brain imaging, tissue slice or single cells [2,8,21-23]. One important advantage is that a fiber-optical probe can be placed in close proximity to the detection tissue with a limited acceptance angle in the receiving fiber, which has been shown to receive more consistent intrinsic optical properties with less scattering artifact in brain slice under osmotic challenges [23]. In addition, the utilization of optical fibers can avoid the necessity of optical focus elements and then reduce the scale of operation space. Consider the mentioned advantages and the low cost of optical fibers, a fiber-guided optical path was adopted in the optical probe design of this study.

In general, optoelectronic study of biological tissue utilizes both the intensity attenuation and the phase shift of scattered light for tissue characterization. The applications of scattering optical signals range from noninvasive recording of functional human brain mapping, image of brain slice, and in-vitro neuronal culture. Recently developed frequency-domain near infrared spectroscopy (NIRS) techniques, i.e. the sinusoidal intensity modulation, now make it feasible to extract both amplitude and phase information from scattering light of brain activity [3,4]. It is known that photon travel time varies in biological tissues according to the length of the path traveled between scattering events and the number of scattering events when light is injected into a rich scattering medium. Thus, optical delay expressed as phase shift has been developed as a useful indicator in macro-scale study designed to monitor the alternation of brain activity states [2-4]. However, most contemporary light scattering approaches of cellular studies emphasize light intensity and scattering angle changes obtained by monitoring of continuous DC illumination before and after altering the physiological status of the target cellular system [9,24-26]. Few studies have investigated changes in both light intensity and phase information of neuronal tissue [27], largely because of the inability to detect small phase shift information in frequency-modulated methods.

The aim of this present study was to develop and test an optoelectronic system which used sinusoidal intensity modulation of the laser source with a miniaturized fiber optic probe which can be placed in proximity to the aimed cell tissues so as to avoid large alignment and focusing devices. Both light attenuation and phase shift were tested in cultured cells whose scattering properties and surface morphology were known to vary in different osmotic media. Near infrared range (650-900 nm) is relatively immune to absorption from water and culture medium, i.e. an "optical window" for biological tissue, and so was employed in the study. The probe was fabricated by binding two parallel optical fibers together, one for guiding the NIR source light and one for capturing the backscattered photons at a limited receiving angle to a detector. Such a design not only reduces the volume and complexity of the source/target/detector system but also limits the diffusive area to only the targeted cells. Experimental setup was designed to confirm the our design which could detect the intensity and phase changes of intrinsic optical backscattering signals from cultured cells undergoing osmotic challenges which is beyond just morphological changes [17,26].

## Methods

### System setup

**Figure 1:**
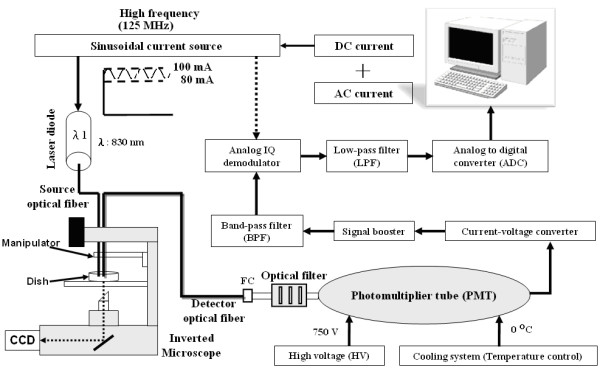
**The overall block diagram of the high-frequency modulation optical system operated with near infrared light source and PMT detector**.

Figure 1 depicts the setup of the developed optical backscattering measurement system. It consists of three major parts: 1) high-frequency intensity-modulated laser light source; 2) light detector; 3) signal processing and data acquisition module. The laser diode was excited by a sinusoidally intensity-modulated current source. The laser light is channeled through an optical fiber to the target cells. The scattered photons, after interacting with the cellular tissue, are captured by the light-detecting optical fiber. Then, to compensate for low backscattering intensity, the captured photons are guided to a highly sensitive photomultiplier tube (PMT) and converted to voltage signal. The signal processing module, including in-phase/quadrature-phase (IQ) circuit, low-pass filter and amplification circuit, demodulates the high-frequency intensity-modulated signal down to low frequency for sampling by the data acquisition module. Finally, the sampled signals are sent to a personal computer for further signal analysis.

**Figure 2:**
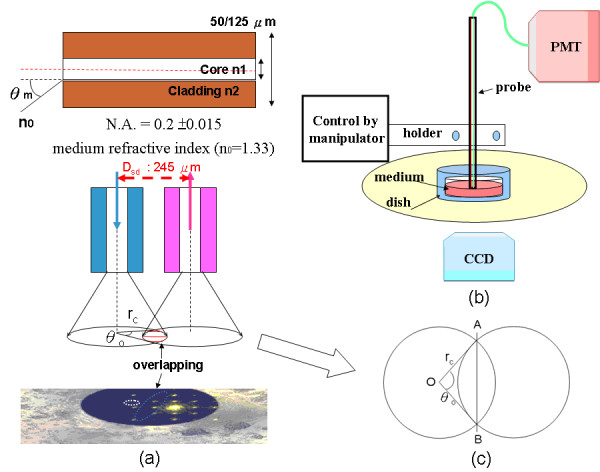
**Schematic diagram of a fiber-optical probe made by binding two parallel optical fibers together for detecting the optical changes of overlapping area of cortical neurons**. (a) (upper part) The half-angle of light cone of a fiber is characterized by the numerical aperture (N.A.). With the half-angle and source/detector distance determined, the minimum distance from probe tip to dish that makes two light cones overlapping can be observed as the detection area. (b) The system setup is designed for manipulating the fiber-optical probe at varied distances from the probe tip to the culturing cells as well as for cellular experiment under varied osmotic pressures. The scattering photons are captured by PMT which can be correlated with the cell coverage of the cellular image capturing by CCD (shown at left lower corner) for calibration curve. (c) Expanded view and labeling of overlapping area for calculating the optical detection region.

The light source was driven by an intensity-modulated current source combining DC current bias from a constant source (PRO800 & LDC8005, Thorlabs, New Jersey, USA) and AC-modulated intensity from a radio frequency (RF) signal generator (E4430B, Agilent, USA) at 125 MHz. The intensity-modulated current drove a laser diode (HL8325G, Hitachi, Tokyo, Japan), producing a wavelength of 830 nm in the NIR region. The amplitude-modulated laser light was guided to the targeted cells via an optical fiber probe made by binding two micrometer-scale optical fibers of 50/125 μm in diameter to form a fiber-optical probe of lateral dimension of 245 μm (figure 2). Such shortened source/detector (S/D) distance design ensures the light intensity focusing in a minimized cellular volume. The fiber-optical probe was attached to a micromanipulator (Olympus MN-151) with 5-μm resolution under an inverted microscope for direct observation of the targeted cells.

The optical detection system is composed of the optical detector module and a cooling system. For scattered light detection, an infrared pass filter (IR720H, ROCOES Electro-Optics, Taichung, Taiwan) was used to reduce the influence of ambient light and permit wavelengths longer than 720 nm to pass through. In consequence, the entire system can be operated in a normally illuminated laboratory environment rather than a dark room. The PMT (R928, Hamamatsu, Japan) with high sensitivity and fast optical responses was adopted. A lab-made cooling system with temperature-controlled Peltier module was designed as a cylinder-shaped case to house PMT closely. A water cooling system was used to accelerate heat dissipation and alleviate thermal noise resulting from unstable temperature. Light detection by the PMT was output as a current signal which was then converted into a voltage signal via a current-to-voltage converter (THS3201, TI, Texas, USA) before signal amplification and processing.

For detecting low optical scattering output, an amplifier with 30-dB gain and a band-pass filter (3-dB bandwidth at 10 MHz) over the range of the selected modulation frequency was utilized to enhance the signal-to-noise ratio (SNR). An IQ demodulator (9010-37, TeleTech, Montana, USA) was used to recover intensity (attenuation) and phase shift information from the I and Q components. The IQ demodulation not only converts the output bandwidth from RF to intermediate frequency but also preserves the intensity and phase information [1]. Finally, the demodulated signal was sampled via analog-to-digital converter (PCI-6143, National Instruments, Texas, USA) at 5 kHz then analyzed offline to derive the intensity attenuation and phase shift change using MATLAB (Mathworks, Massachusetts, USA).

### Validation experiments

#### Determination of optimal measuring distance

With reference to figure 2(a), an optical fiber has conical light diffusion area such that the distance between the tip of the optical fiber and the surface of the cell-culturing dish is a primary determinant of the area of light diffusion. In our probe configuration, the parallel optical fibers of the emitting and receiving probe have overlapping diffusion areas so that the probe/target distance has a strong influence on the detection area and the amount of backscattered light that is detected. In addition, the interprobe distance (distance between the emitting and receiving fibers), the specific characteristics of the optical fibers and the target medium should also be considered. By incorporating the numerical aperture (NA) and medium refractive index into equation (1), in theory, one can calculate the refraction angles of the emission and reception light cones from the trigonometric relationships between fiber-optical probe and surface of culture dish as(1)

where *θ*_*m *_is the refraction angle between fiber and medium. The refractive indices of medium, fiber cladding and fiber core are n_0_, n_1 _and n_2, _respectively. The overlapping area can be calculated using equation (2) by doubling the difference between triangle and fan shape region within points O, A and B in figure 2(c).(2)

where *r*_*c *_and *θ*_*o *_are the radii of the light cone on surface of culture dish and the radian between the two intersecting edges, respectively. The values of *r*_*c *_and *θ*_*o *_are determined by *θ*_*m*_, the probe/target measuring distance. *D*_*sd *_is the intercentral distance between emitting and receiving probes. Theoretically, the best distance from the tip of probe to the dish surface involved two light cones begins overlapping at 639.4 μm. This can be derived from theoretical computation based on NA of 0.2 and medium refractive index (n_0_) of 1.33 with a refraction angle of 8.65° from equation (1).

With fixed geometrical distance and angle between sensing and detecting fibers, increasing the distance between the probe tip and the surface of culture dish will increase the overlapping area between the emitting and detecting cones. Although the increase in the overlapping area can gather more scattered photons, the longer distance also results in higher attenuation of the receiving light. Thus, a practical test to determine the optimal measuring distance between the probe and the culture dish should be performed. For this test, we progressively moved the fiber-optical probe up from the surface of dish with the aid of the micromanipulator and simultaneously recorded the scattered light. Then, the changes in intensity and phase of recorded scattering light were compared at different measuring distances.

#### Relationship between cellular volume and scattering light intensity

For comparing the light scattering outputs of different cell cultures, a calibration process which correlates the linear relationship between cellular volume (density) and scattered light intensity is needed to make sure that the scattering output mainly depends on cell volume under the fixed measuring distance. Figure 2(b) depicts the experimental setup for capturing the cell image of the targeted area and the scattered output, for analyzing the relationship. It is known that greater cell density produces less optical transmittance as well as induce more scattering events so as to increase the scattering intensity [10]. The charged coupled device (CCD) camera mounted on microscope was used for capturing cellular image. The optical fiber was first aligned to the center of the CCD image. Without altering the focus of the microscope and the optical fiber, for deriving the relation curve, a series of randomly selected cellular images and the corresponding optical scattering output were taken while moving the microscope stage horizontally. For obtaining the area of cell coverage, the cellular images were converted into binary forms via image processing techniques of edge detection, linking and hole-filling. Then, a graphic user interface (GUI) programmed in MATLAB was used to calculate the cell coverage within the source/detector overlapping area by counting the pixels of the binary cell image. The relation curve, the linear regression line between the cell coverage and the measured scattering light intensity, was derived for the subsequent cellular experiments.

#### Optical scattering responses to osmotic shocks

After the desired optical distance to cells and the relationship of scattering light to cell density were obtained, the following experiments were performed to observe the IOS of cells in different states that were produced by experimentally changing the osmotic pressure in the culture medium. The experimental protocol was approved by the National Cheng Kung University Animal Care and Use Committee. Cortical neurons dissociated from newborn Sprague-Dawley (SD) rats (born within one day) were cultured on dishes of 3 cm in diameter. Each culture dish was filled with 2 ml of isosmotic (350 mOsm/kg H2O) culture medium which was composed of neurobasal medium (GBICO, NEUROBASAL™ Medium; Cat. No. 21103-049) containing B27 serum-free supplement (GBICO, Cat. No. 17504-044). The B27-suplemmented neurobasal medium was required to maintain the neurons' viability and growth which was replaced every 2-3 days. Except medium replacement and essential viability observation, the culture dishes were kept in an incubator with the temperature maintained at 37°C and air at 5% CO_2_.

For varying the osmotic conditions in the culture medium, hyposmotic culture was obtained by adding distilled water (DI water) and hyperosmotic culture was obtained from isosmotic baseline by adding solution of varied volume of 10× phosphate-buffered saline (PBS) consisting of NaCl, KCl, Na_2_HPO_4_, KH_2_PO_4_, H_2_O. For dishes with cultured cortical neurons, the osmotic shocks were applied gradually with hyperosmotic (+20, +40 and +60 mOsm) and hyposmotic (-20, -40 and -60 mOsm) challenges. Culture dishes with medium only filled with hypo-/hyperosmotic solutions were prepared as control to ensure that the osmotic solution would not affect the measured scattering properties of cultured neurons.

For each osmotic change experiment, time-course measurement of the scattered light was performed. For each measurement, the recording period lasted 7 minutes, beginning with 2 minutes of isosmotic condition followed by 3 minutes of hyper or hyposmotic shock, and finally ending with 2 minutes of isosmotic condition, recovering by adding hypo-/hyperosmotic solution. Since the distribution and amount of cells could not be precisely controlled, a normalization process of the measured scattering output was needed for comparison between dishes.

### Data analysis

From the optical outputs of the dish with medium only and those with cells under varied osmotic shocks, the amplitude and phase of scattered light were derived from the IQ demodulator according to equations (3) and (4).(3)

where *I*_*dc *_and *Q*_*dc *_are the I and Q components from the IQ demodulator. However, the absolute amplitude change and phase shift might vary among individual dishes. Thus, the dish area with more even distribution of cells was chosen. From the targeted cells, the amplitude and phase of backscattering light recorded from 20 seconds before osmotic shock were averaged as a reference. The relative values were obtained by calculating the differences between the measured amplitude (A_exp_) and phase (P_exp_) with those from the reference, A_ref _and P_ref_. The relative amplitude, ΔRa, was obtained by further normalizing by the pixel count (N_p_) of the cell coverage within the overlapping, as indicated in equation (5). The phase shift, ΔRp, is the difference between the osmotic-induced signal and the reference, P_exp _and P_ref_, respectively.(5)

## Results

### Measurement of high-frequency intensity-modulated near infrared signal

Validation of the current fiber-guided optical system was first tested by perpendicularly aiming the parallel-fibers probe at a culture dish with medium only. A high performance oscilloscope with sampling rates up to GHz was utilized to observe the driving current and the output of the PMT response to incident photons. The emission power was 42.75 mW (at 1.462 V) and reception power was measured at 64.39 μW (at 56.68 mV). This measurement demonstrated that the presented parallel optic fiber probe scheme received signal had sufficient SNR for use in the following calibration and cellular experiments.

### Scattering properties with probe at different measuring distances

**Figure 3:**
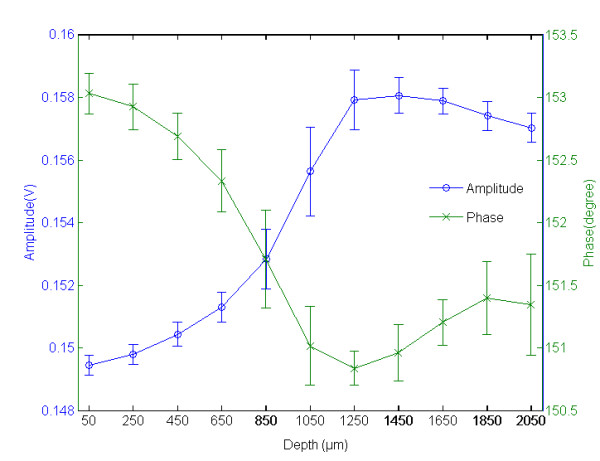
**The relationship between the averaged intensity and phase data measured at various distances from the tip of fiber-optical probe to the surface of culture dish**. The sigmoid-like trend in the intensity (-o-) of optical output is found with the increase of measuring distance, which peaks around 1250 μm. Opposite trend can be observed in the phase information, with a minimum around 1250 μm.

To dertermine the optimal distance between the tip of probe to the dish surface, the scattering outputs at different probe-to-dish distances ranging from 50 to 2050 μm at stepwise increments of 200 μm were measured. We can observe experimentally the tradeoff between the increase in overlapping area and the decrease in optical intensity. The amplitude and phase data of five sampled medium-only dishes were averaged, as shown in figure 3. We observed that the received optical intensity remained at low level at probe-to-dish distances shorter than 450 μm but abruptly increased as the distance exceeded 650 μm. The optical outputs reached a peak value around 1250 μm followed a slightly decreasing plateau up to 2050 μm. In contrast to the increasing trend in optical intensity, the phase shift exhibited a decreasing trend to a minimum around 1250 μm and then gradually increased again. Thus, a probe-to-dish distance of 1250 μm, with an overlapping detection area about 46309 μm^2^, was chosen for the following osmotic challenge experiments.

### Relationship between backscattering outputs and volume of cells

**Figure 4:**
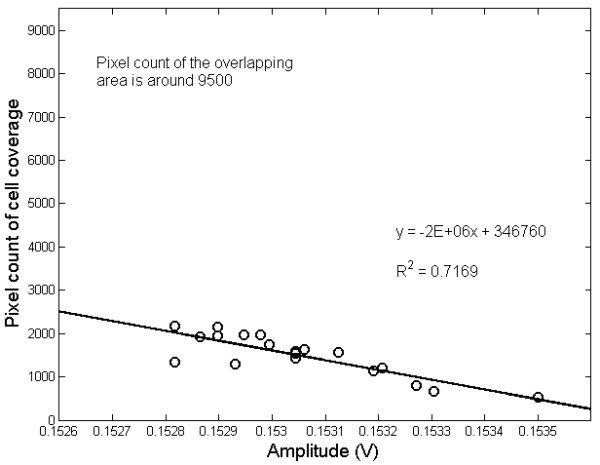
**The linear relationship between the mean light intensity versus the cell coverage of cellular image within the overlapping detection area**.

To obtain the relationship between backscattering output and volume of cells, nineteen samples of scattered light intensity, each of 20 seconds duration, were randomly collected and averaged from the cell culture dish. For each sample, the corresponding cellular CCD images were captured to determine the area coverage of the target cells within the overlapping detection area of the fiber-optical probe. Figure 4 shows one example of the relationship between the scattering output and cellular volume obtained from the mean light intensity and the pixel count of cell coverage. The 46309 μm^2 ^of overlapping area was also converted to image pixels about 9500 to allow comparison with the numbers on the ordinate axis in Figure 4. A negative correlation with R-squared value of 0.7169 was found in our perpendicular backscattering scheme. From the scatter plot, we can observe two outlier points deviated from the regression line in higher pixel image representing the area of larger coverage of cells which might be the condition of over-stacked cells causing larger variation in optical backscattering measurement. Thus, appropriate selection of cell density before the preparation of a cell culture is needed, which was taken into consideration in the next experiment of cells under the influence of varied osmotic media.

### Light scattering responses on culturing cells

Before testing cells modulated by varied osmotic media, the backscattering properties of the medium alone, i.e. PBS and DI water without cultured cells, were tested. Table 1 lists the mean scattering intensity and phase value of five samples under conditions ranging from isosmotic to hyposmotic by adding DI water as well as conditions from isosmotic to hyperosmotic conditions by adding 10× PBS. The osmotic changes of the two extreme conditions in our cell culture experiment, ± 60 mOsm, were tested. For all conditions, the light intensity remained steady except for small variation with no statistically significant change. Similar statistically insignificant variations were observed for phase measurement over the hypo-to-hyperosmotic range of conditions.

**Table 1:** The characteristics of scattering light under various osmotic solutions without cultured cells

Dish (n = 5)	Isosmotic	Hyposmotic -60 mOsm (add DI-water)	Isosmotic	Hyperosmotic +60 mOsm (add 10× PBS)
Amplitude	0.1563 ± 2.19 × 10^-5^	0.1563 ± 2.46 × 10^-5^	0.1563 ± 2.15 × 10^-5^	0.1563 ± 1.80 × 10^-5^

Phase	151.0529 ± 0.0042	152.0970 ± 0.0075	151.0529 ± 0.0041	151.0677 ± 0.0049

**Figure 5:**
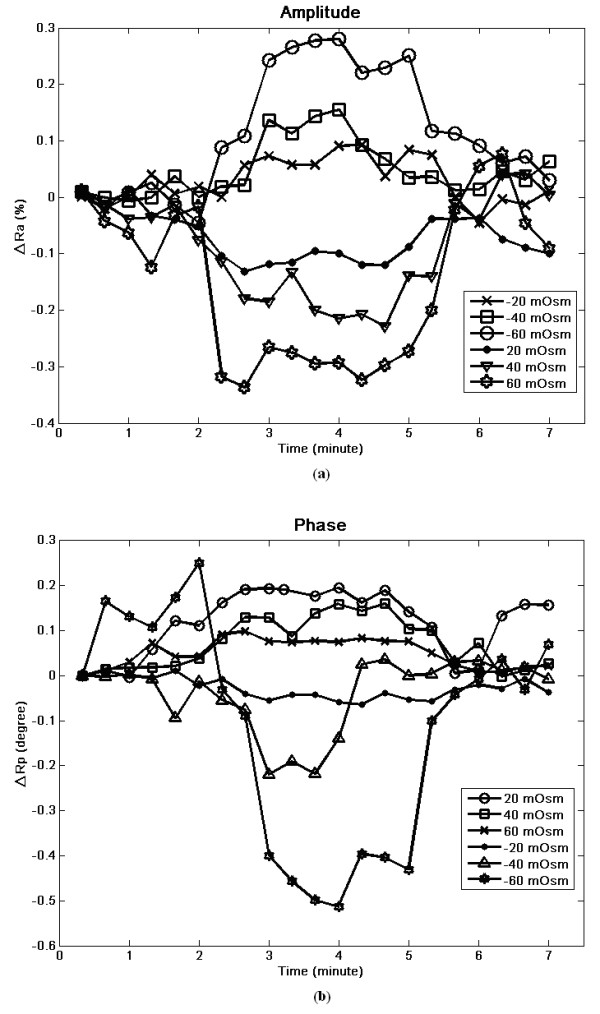
**The changes of scattering light in response to a 7-min exposure to ± 20, ± 40 and ± 60 mOsm of osmotic challenges**. The time-course plots of (a) the relative amplitude (ΔRa) and (b) relative phase shift (ΔRp) under different osmotic conditions. The negative ΔRp indicates the leading in phase compared to that of cells before osmotic challenge.

For backscattering testing of cultured cells exposed to osmotic shock, each of the six osmotic conditions was averaged in 3 culture dishes. The recorded data were first normalized by the cell-free area of each dish and adjusted by the pixel count, N_p_, in equation 5. Figure 5 shows the intensity and phase changes during 7 minutes of data, covering the time from isosmotic to either hyperosmotic or hyposmotic conditions. Clearly, there are regular changes in light intensity in response to the changes in osmotic conditions. Compared to the isosmotic condition, the backscattering intensity increases under the influence of low osmotic pressure. On the contrary, the intensity of backscatter decreases with the shrinkage of cells as a result of increase in osmotic pressure. However, the phase shift changes did not follow those observed for intensity. As seen in figure 5(b), the phase shift changes were greater under hyposmotic conditions relative to those under hyperosmotic conditions. Notably, injecting balance solution to the previously osmotically shocked cultures at the 5th minute resulted in both intensity and phase shift changes returning to close to their initial values, indicating the recovery of cell volumes.

**Figure 6:**
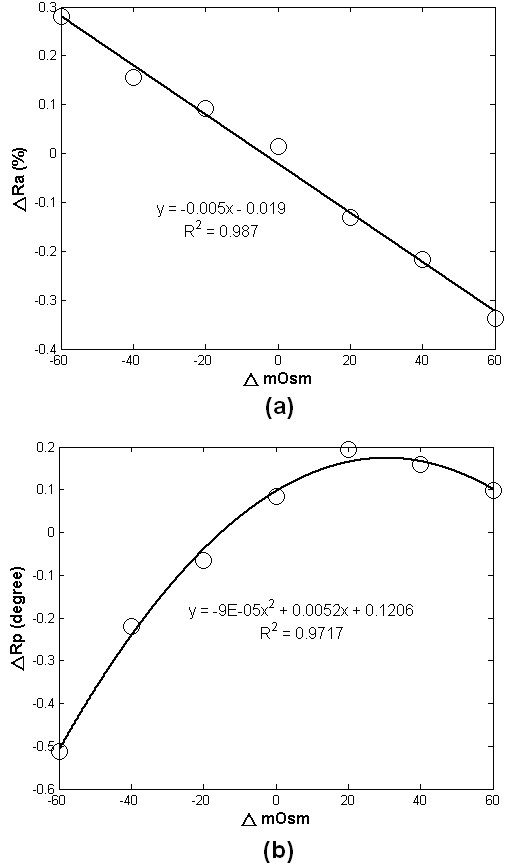
**The largest change in light intensity and phase shift to the different levels of osmotic change**. (a) shows an inverse relationship between osmolality and relative intensity (ΔRa) (b) The relative phase shift (ΔRp) decreases with the decrease of osmotic pressure but reaches a plateau at high osmotic pressure.

Figure 6 summarizes the relationship between the averages of peak changes in intensity and phase versus the osmotic changes. The relationship between ΔRa and Δosmolality shows an inverse but highly linear (R^2 ^= 0.987) trend over our tested range of -60 to + 60 mOsm. On the other hand, the relative phase change, ΔRp, exhibited a positive but nonlinear relationship relative to osmotic change, as plotted in figure 6(b). The maximum phase shift, in absolution value, increases with the decrease of osmotic pressure that induces cell-swelling. A plateau is reached at high osmotic pressure with the maximal possible shrinkage in the cells.

## Discussion

A fiber-guided backscattering optical system with relatively low output of 0.1 mW on a circular spot area of 215 μm in radius or 46309 μm^2 ^of overlapping between source illumination and detection area has been described and experimentally verified as a useful tool for observing and monitoring the changes of IOS of cells under various osmotic solutions. The presented design uses parallel optical fibers in close attachment as an optical probe of compact design. One possible way of more compact probe design is to utilize a 3-port optical circulator (OC) to deliver and collect light from the same optical channel. However, high attenuation in receiving light of OC would aggravate the detection sensitivity of already low level of backscattering light. The overlapping of the emission/reception cones is not a factor in system with OC. However, it still requires height calibration because it could be too close with limited detection area and too far with undetectable scattering intensity. Compared to our fiber-optical probe design, most previous approaches have employed focusing or scanning lens and higher optical output (several times of our level) for intrinsic optical properties studies [9,14,25]. Although higher optical power covers larger area, excessive light applied to a group of neuronal cells also induces a higher background signal and makes the detection of small changes in scattering very difficult. There is further potential damage to the cell tissues. Utilization of low intensity of emission guided by fiber-optical probe might alleviate the high background problem, making the small changes in backscattering light easier to be detected, as demonstrated in this study.

It was determined that the distance from the fiber-optical probe to the cell target is a key factor as a result of the changes to the overlap of the optical fields of the source and detection fibers. The probe/target distance should therefore be optimized to produce optimal optical output relative to the size of the detection area. The trend of experimental results, as depicted in figure 3, is close to the theoretic calculation derived from the optical emission aperture, refraction coefficient and emission distance from probe to cells. The experimental results of overlapping area can arise at a distance of 650 μm, which is very close to the theoretical value of 655 μm, remembering that there is ± 10 μm variation between the neighboring ports of the fiber-optical probe. Although a longer measuring distance can increase the overlapping measurement area, it also attenuates the optical output. Our experimental results show that the recorded backscattering intensity attenuates progressively for measuring distance between 1250 and 2050 μm.

The IOS of opposite polarities, increased or decreased under hypo/hyper osmolality solution, were commonly reported by investigators [14,23]. The sign discrepancy mainly originates from different setups of optical systems, e.g. reflection type versus scattering type. However, even the same scattering type of optical signal, the experiment process, e.g. single or multiple scattering in the cellular or brain tissue, reflection from the substrate, and potential artifacts associated with scattering, might result in different polarity measures [23]. Thus, it is essential to have validation experiments to test a new setup of optical system for IOS measurement under challenges, e.g. osmotic shock in this study. Since the cellular image stands for the light transmittance distribution of the object, the area of denser cellular population in an image exhibits higher value in pixel count of binary image in comparison with those with less cell volume (looser case). It is expected that an area covered by more cells produces more backscattered light. Thus, a positive regression line between covered cell volume and backscattered light should be found, especially for oblique backscattering [14,25,26]. However, an opposite to the expectation of positive relationship between cell volume and backscattering intensity was found, as seen in figure 4, which should be attributed to the probe scheme.

Because our fiber-optical probe is almost perpendicularly aimed at the dish surface, the parallel probing in this study could collect not only backscattering but also the mirror reflection from cell-free dish surface. This design is different to the previous backscattering studies which usually design a specific angle between source and detection to prevent receiving mirror-reflected or forward-transmitted light [14,25,26]. When the probe moved onto a cell-free area, the mirror reflection was dominant. However, as the overlapping area moved to cultured cells, the decrease in reflection as well as the increase in scattering reduced the obtained light intensity. Thus, the scheme of parallel probing could explain the results of a higher light intensity obtained in the case of looser cell population and a lower one obtained a denser case. However, in thick tissue such as a brain slice or in-vivo animal model, the absorption/scattering of cellular organelles would be the dominant factors. Effect of the multiple scattering and absorption would accelerate the light attenuation and contribute the random/isotropic distribution of backscattered photons. Such effect shortens the propagation range of backscattering and hence decreases the probability that backscattered photons reach detector. This condition is like the case of multiple scattering in functional NIRS studies [2-7,11] where the experimental scheme usually utilizes detector with large reception area and contacts the probe on tissue tightly to increase ability of photon detection.

The results of the osmotic shock experiments confirmed the presented system as a suitable tool for monitoring IOS changes in cultured cells under challenges. Backscattering shows high linearity (R^2 ^= 0.987) between osmotic modulation and optical response, as shown in figure 6(a), demonstrating the ability to detect IOS changes due to tiny volumetric changes. The phenomena of regulatory volume decrease (RVD) and regulatory volume increases (RVI), i.e. the volume changes under the influence of osmotic solution, were clearly observed in previous osmotic challenge experiments [14,26]. The onset of change in optical output due to osmotic change and the recovery behavior of cells were also clearly recorded. For intensity, the resolution sensitivity of -0.3 ~ +0.3% found for this study is comparable to those of previous backscattering studies [14,26]. However, the direction of change, i.e. increased backscattering intensity with decrease in osmotic pressure, is just opposite to those observed from these prior works [14,25,26]. This disparity between our approach and the others is most likely explained by the same reason, the negative relationship between the area of cell coverage and light intensity shown in Figure 4.

For the hyposmotic condition, the water influx induces cell swelling which would smooth the cell morphology and decrease the refraction index of cytosol. Therefore, the almost perpendicular diffused photons would result in less scattering on even or less wrinkled cell membrane/medium interfaces, which in turn means higher probability to directly penetrate into cells and then be more reflected from the cell membrane-dish surface. On the contrary, hyperosmotic challenge causes cell dehydration, more folding in cell membrane and denser cytosol. These conditions lead to the increase in medium/membrane scattering but less intracellular collision, so that less photons can unimpededly travel to dish surface through a scatter-free path for more mirror reflection.

In this study, the changes in phase of backscattering light were found in the distance determination experiment as well as in the osmotic shock conditions. However, phase shift change was not as linear as the change of light intensity. Under the hyposmotic solution, swelled cells not only extended the lateral cell coverage but also increased the cell height, which would elongate the travelling time in cytosol. In addition, the water influx during cell swelling also decreased refractive index. The reverse trend should be observed when cells are bathed in high-osmolality solution. However, a rather linear phase shift was found in hyposmotic solution compared with that in hyperosmotic one. The phase shift plateau reached in the hyperosmotic solution is possibly due to a limitation in possible cell shrinkage because of a minimum space needed for organelles and nucleus within the cell, thus preventing unlimited shrinkage. Therefore, the nonlinear relationship between phase shift and osmotic challenge should be considered for utilizing phase information in cell status monitoring.

## Conclusion

A high frequency modulated near infrared optical system parallel-fiber probe to observe the optical properties of cultured cells, i.e. IOS, via backscattering properties has been demonstrated. The simple design of dual-fiber probe can constrain the region of interest of cultured cells and thus limit the influencing factors of backscattering. One dominant factor related to the measurement sensitivity is the distance between the fiber-optical probe and cells in which an optimal distance can be determined from calibration experiment. The relationship between backscattering and cortical neurons under varied osmotic challenges was performed as a validation test. Experimental results show that the intensity of backscattering was found to be inversely proportional to cell coverage due to the osmotic changes. However, the phase shift exhibited a nonlinear feature and reached a plateau at hyperosmotic solution. The utilization of fiber-guided NIR light provides compact and mechanically flexible setup as a new modality for observing IOS of neuronal cell in backscattering ways. However, further studies are necessary to better understand the thickness factor of cultured cells or tissue layer and their relationship to the nonlinear phase-shift observed in this study. Our ongoing project is to combine NIR backscattering measurement device and a multielectrode array (MEA) to simultaneously monitor both optical signal and electrophysiological data, including action potential and impedance. Neuronal cells cultured in different conditions, such as ischemic stroke condition or under electrical stimulation, would be essential for further understanding the scattering properties of neuronal cells for the future application of cell-based biosensor and drug screening. Also, the backscattering properties of in-vitro cultured cells could provide foundation for understanding the IOS properties of neuronal activity of brain in-vivo.

## Competing interests

The authors declare that they have no competing interests.

## Authors' contributions

All authors have contributed to and approved the final manuscript. CHH and ETL conceived and designed the study. CHH, GCC, ETL and YJL developed the methodology and acquired the data. CHH, ETL and YJL conducted statistical analysis. JJJC advised with study design and participated in writing the manuscript.
